# Novel signature fatty acid profile of the giant manta ray suggests reliance on an uncharacterised mesopelagic food source low in polyunsaturated fatty acids

**DOI:** 10.1371/journal.pone.0186464

**Published:** 2018-01-12

**Authors:** Katherine B. Burgess, Michel Guerrero, Andrea D. Marshall, Anthony J. Richardson, Mike B. Bennett, Lydie I. E. Couturier

**Affiliations:** 1 School of Biomedical Sciences, The University of Queensland, St Lucia, Queensland, Australia; 2 Marine Megafauna Foundation, Truckee, California, United States of America; 3 CSIRO Oceans and Atmosphere Flagship, EcoSciences Precinct, Dutton Park, Queensland, Australia; 4 Fundacion Megafauna Marina del Ecuador (Proyecto Mantas Ecuador), Quito, Ecuador; 5 Centre for Applications in Natural Resource Mathematics, The University of Queensland, St Lucia, Queensland, Australia; 6 Université de Bretagne Occidentale, UMR 6539 LEMAR (IRD/UBO/CNRS/Ifremer), Laboratoire des Sciences de l’Environnement Marin, IUEM, rue Dumont d’Urville, Plouzané, France; University of Illinois, UNITED STATES

## Abstract

Traditionally, large planktivorous elasmobranchs have been thought to predominantly feed on surface zooplankton during daytime hours. However, the recent application of molecular methods to examine long-term assimilated diets, has revealed that these species likely gain the majority from deeper or demersal sources. Signature fatty acid analysis (FA) of muscle tissue was used to examine the assimilated diet of the giant manta ray *Mobula birostris*, and then compared with surface zooplankton that was collected during feeding and non-feeding events at two aggregation sites off mainland Ecuador. The FA profiles of *M*. *birostris* and surface zooplankton were markedly different apart from similar proportions of arachidonic acid, which suggests daytime surface zooplankton may comprise a small amount of dietary intake for *M*. *birostris*. The FA profile of *M*. *birostris* muscle was found to be depleted in polyunsaturated fatty acids, and instead comprised high proportions of 18:1ω9 isomers. While 18:1ω9 isomers are not explicitly considered dietary FAs, they are commonly found in high proportions in deep-sea organisms, including elasmobranch species. Overall, the FA profile of *M*. *birostris* suggests a diet that is mesopelagic in origin, but many mesopelagic zooplankton species also vertically migrate, staying deep during the day and moving to shallower waters at night. Here, signature FA analysis is unable to resolve the depth at which these putative dietary items were consumed and how availability of this prey may drive distribution and movements of this large filter-feeder.

## Introduction

Manta rays (formerly in the genus *Manta* [1]) are among the largest elasmobranch fishes and currently comprise two recognised species, the reef manta ray *Mobula alfredi (previously Manta alfredi)* and the giant manta ray *Mobula birostris* (previously *Manta birostris*). Manta rays occur in mid- to low latitudes and while both species move over hundreds of kilometres [2, 3], they often aggregate at particular sites throughout their respective home ranges [4, 5]. The traditionally held view is that manta rays feed on surface zooplankton during daylight hours at these aggregation sites, and that aggregative behaviour therefore is linked with local-scale food availability [4, 6]. Both *M*. *alfredi* and *M*. *birostris* have been observed feeding in surface waters [7, 8], with *M*. *alfredi* commonly seen feeding at aggregation sites, whereas for *M*. *birostris* surface feeding at aggregation sites is more unusual [5]. For aggregation sites where surface feeding activity has been documented, it is unclear how important this mode of foraging is to an individual’s overall dietary intake.

Determining drivers of *M*. *birostris* aggregative behaviour and movement patterns, and how these are linked to foraging opportunities, helps identify where this vulnerable species is most susceptible to direct or incidental capture in fisheries [3], or subject to other unsustainable anthropogenic pressures such as poorly managed ecotourism activities [9].While stomach contents analysis has provided some useful dietary information about *M*. *alfredi* [10], these data provide a ‘snap-shot’ of recent feeding behaviour and may or may not be representative of feeding ecology more generally. In addition, obtaining specimens of rare and internationally protected species, such as manta rays, is unethical or at best, challenging as samples have to be obtained from markets, therefore alternative approaches are needed. The application of non- lethal molecular methods, wherein small muscle tissue biopsies can be taken and analysed, has the ability to provide information on assimilated dietary intake over long time-frames [11]. The recent application of such molecular methods for reef manta rays and whale sharks has provided insight into their feeding ecology [12]. These studies suggest that surface zooplankton may comprise a small proportion of dietary intake, with the majority of their diet sourced from deeper or demersal sources [13–15].

Aggregation sites for *M*. *birostris* recently discovered off mainland Ecuador provide access to what appears to be the largest identified regional population of this species [16, 17]. These sites provide a unique opportunity for observation of behaviour and collection samples for molecular analyses from this largely elusive species. Signature fatty acid (FA) analysis provides a way to assess trophic interactions as marine primary producers can usually be identified via the presence, absence or combination of certain FAs [18]. Omega-3 and omega- 6 polyunsaturated FA (PUFA) are considered physiologically vital in marine fishes as they are constituents of complex lipids and can influence growth, reproduction and survival [19, 20]. PUFAs are thought to be transferred and concentrated throughout the food web [21], inducing a potential bottom-up control on animal population (i.e., influence of lower trophic forms on the higher ones) [22, 23]. Important PUFAs include: docosahexaenoic acid (DHA), eicosapentaenoic acid (EPA) and arachidonic acid (ARA). DHA influences cell membrane characteristics such as fluidity or permeability, while EPA and ARA are precursors of antagonist eicosanoids, i.e., hormone-like compounds that are produced in response to changes in fatty acid composition of available food resources [24, 25]. Most marine fishes are unable to synthesise PUFAs due to the lack of elongation and desaturation enzyme systems, and need to acquire them from their diet [24]. As these PUFAs are a direct consequence of diet, they can be used as a chemical biotracer of food origin.

In this paper, we present the first FA and lipid class (LC) profiles of *M*. *birostris* and surface zooplankton found off mainland Ecuador. The aim of this study was to provide insight into the long-term (~12 months [26, 27]) assimilated diet of *M*. *birostris* by comparing the FA profiles from muscle tissue of this species and surface zooplankton at a coastal aggregation site off mainland Ecuador. Based on previous stable isotope work, which found that *M*. *birostris* off mainland Ecuador gain the majority of their diet from mesopelagic sources [15], we predict that: (1) the FA profile of *M*. *birostris* from this region will be representative of mesopelagic dietary sources and therefore, (2) the FA profile of *M*. *birostris* will be largely different to the FA profile of surface zooplankton, assumed to be rich in PUFA [13], collected from the same region.

## Methods

### Sample collection

*Mobula birostris* muscle samples and surface zooplankton were collected at Isla de la Plata (1.2786° S, 81.0686° W) and Bajo Copé (1.81706° S, 81.06362° W), Ecuador. A total of 49 *M*. *birostris* biopsies, each from a different, visually identified individual, were collected on SCUBA during their seasonal aggregation period (August–October [17]) over several years (2012, n = 11; 2013, n = 9; 2014, n = 29) by use of a modified hand spear. The sex of *M*. *birostris* individuals was determined through the presence (male) or absence (female) of claspers [28]. Feeding behaviour was recorded when *M*. *birostris* were engaged in continuous ram-feeding on zooplankton patches, with an open mouth, visible gill rakers and unrolled cephalic fins.

Surface zooplankton was collected via horizontal surface tows with a 200 μm mesh plankton net (50 cm mouth diameter) for 5 minutes at an average speed of 0.5–1 m s-1. Samples were collected during manta ray feeding (n = 3) and non-feeding events (n = 29), and at regular intervals throughout the day in order to cover the full tidal cycle. Temperature was recorded *in situ* as the average temperature between 0–25 m depths using a dive computer (Suunto D6i) and reported to the nearest whole number.

All *M*. *birostris* and zooplankton samples were placed on ice immediately after collection and then stored at -18°C, or on dry ice, until required for FA analyses. This study was conducted in accordance with the University of Queensland Animal Ethics approval number SBS/319/14/ARC/EA/LEIER. Field work was done with approval from the Ministerio del Ambiente del Ecuador and the Machalilla National authorities, with Proyecto Mantas Ecuador, the NAZCA Institute for Marine Research and Fundacion Megafauna Marina del Ecuador under permits: 008 RM-DPM-MA (2012), 011 AT-DPAM-MAE (2013) and 009 AT-DPAM-MAE (2014).

### Lipid extraction

Muscle biopsies from *M*. *birostris* (n = 11) were freeze-dried and immediately lipid extracted following a 1-day modified Folch method, or were processed wet and lipid extracted using a 3-day Folch method (n = 37) (S1 Appendix) [29]. The total lipid extract from both methods was dried under a stream of nitrogen gas, weighed, and stored at −18°C until further analysis.

### Fatty acid signature analysis

An aliquot of the total lipid extract (LE) was trans-methylated to produce fatty acid methyl esters (FAME) (S1 Appendix) [30]. For gas chromatography (GC) analysis, an Agilent Technologies 7890B GC equipped with a non-polar Equity™-1 fused silica capillary column (15 m × 0.1 mm internal diameter, 0.1 μm film thickness), a digital electrometer, a split/splitless injector and an Agilent Technologies 7683B Series auto-sampler was used. The carrier gas was hydrogen with the generator set at 620 kPa. Samples were injected in split-less mode at an oven inlet temperature of 250°C. After injection, samples were held at 50°C for 1 min, then oven temperature was raised to 230°C at 2.5°C min−1. Gas Chromatography/Mass Selective Detector ChemStation software (Agilent Technologies, Palo Alto, California) was used to quantify FAME peaks. Peak identities were then confirmed with a Thermo Finnigan GCQ GC-MS system (Finnigan, San Jose, California). Fatty acids were expressed as area percentage of total FA (% FA).

### Lipid class profiles

Lipid class (LC) profiles were determined for a representative subset of *M*. *birostris* (n = 10) and zooplankton (n = 6) samples. The total LE from each sample along with a standard solution containing known quantities of common lipid classes; wax esters (WE), triacylglycerols (TAG), free fatty acids (FFA), sterols (ST) and phospholipids (PL) was spotted in duplicate on chromarods. The chromarods were then developed for 25 min in a polar solvent (hexane:diethyl-ether:acetic acid, 60:17:0.1, by volume). Chromarods were oven-dried at 100°C for 10 min and then immediately analysed with an Iatroscan Mark V TH10 thin layer chromatograph with a flame ionization detector, which had been previously calibrated for each lipid class. Peak identification was by comparison with sample retention times in relation to the standards and peak areas quantified using SIC-480II Iatroscan™Integrating Software v.7.0- E (System Instruments, Mitsubishi Chemical Medicine). Predetermined linear regressions were used to identify peak areas that were then transformed to mass per μl spotted.

### Data processing and statistical analyses

For comparisons among *M*. *birostris* and surface zooplankton FA profiles, only FAs in concentrations of >1% of the total FA profile were used in analyses. Non-parametric multi- dimensional scaling (nMDS) was used to visually determine relationships of groupings within and between *M*. *birostris* and surface zooplankton clusters. Similarity percentage analysis (SIMPER) was used to identify the contribution of each FA to the observed similarity between and within groups. Classical hierarchal cluster analysis applied to the nMDS plot to show the clustering and % similarity of identified groups. A principle component analysis (PCA) was applied to FA profiles to explore similarities between *M*. *birostris* and their hypothesised surface zooplankton prey. FA profile data was untransformed with a non-parametric Bray- Curtis similarity matrix, with non-metric multi-dimensional scaling plots (nMDS) used to visualise groups within *M*. *birostris* and surface zooplankton samples. All analyses used PAST v3.12 software [31] and unless otherwise stated, all data are reported as mean and standard deviation.

## Results

When *Mobula birostris* were present during the sampling periods of August to October in 2013, and August to September 2014, surface feeding was observed on 8 of 190 research dives.

### Manta ray fatty acid profile

**Fig 1:**
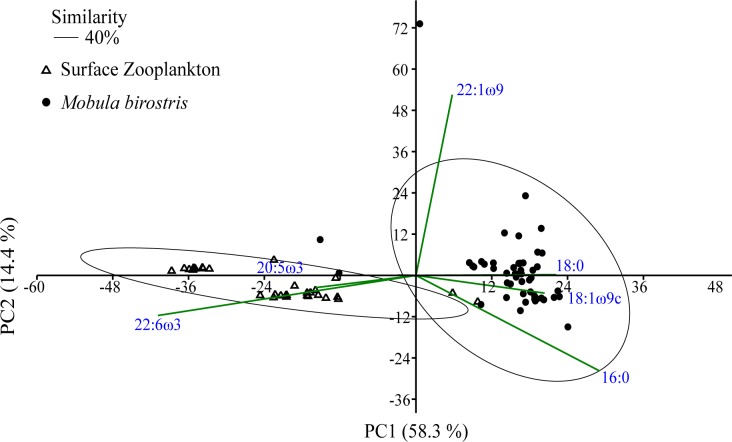
The first and second principle components of *Mobula birostris* and surface zooplankton signature fatty acid (FA) profiles (including all FAs >1% total FA), sampled from off mainland Ecuador. Similarity clusters (40%) are indicated as well as fatty acids contributing most to the separation on the axes (eigenvector coefficient > [0.3]).

There was a significant difference between the FA profiles of *M*. *birostris* and surface zooplankton (ANOSIM, *R* value = 0.8226, p < 0.0001) and an overall average similarity of 39.8%. Largest contributions to dissimilarities were DHA (23.8%,), 16:0 (14%), 18:0 (13.2%) and 18:1ω9c (11.3%) (SIMPER) (Fig 1).

**Fig 2:**
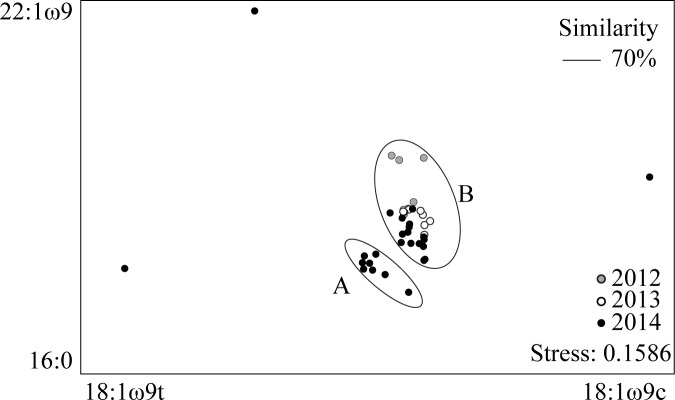
Non-metric multi-dimensional scaling plot of *Mobula birostris* fatty acid profiles (Bray-Curtis Similarity Index). Fatty acid labels represent the main coefficients (> 0.5) contributing to each axis. Clusters A and B refer to two distinct FA profile groups of *M*. *birostris* as revealed by cluster analysis at 70% similarity.

A total of 34 FAs were detected in *M*. *birostris* samples. There was no significant difference between FA profiles of *M*. *birostris* samples that were freeze-dried and extracted with the 1-day method in comparison to wet samples extracted with the 3-day method (ANOSIM, *R* value = 0.109, p =  0.1). The FA profiles of *M*. *birostris* did not significantly differ among years (ANOSIM, *R* value = 0.04753, p = 0.27) (Fig 2, S2 Table) or months (ANOSIM, R = 0.001537, p = 0.45). The average similarity based on the Bray-Curtis similarity measure between *M*. *birostris* samples collected in 2012 and 2013 was 77.6% (SIMPER). There was less similarity between *M*. *birostris* samples collected in 2012 and 2014 (62.5%) than those in 2013 and 2014 (68.1%) (SIMPER).

*M*. *birostris* FA profiles were dominated by saturated fatty acids (SFA) (61.4 ± 11.2%) followed by long chain monounsaturated fatty acids (LCMUFA) (32 ± 11.4%) of which 18:1ω9 isomers were most abundant (Table 1). The principal PUFA was ARA accounting for ~2% of total FA profile (Table 2).

**Table 1:** Fatty acid (FA) profiles, reported as percentage proportion of total FA, for *Mobula birostris* and surface zooplankton sampled off mainland Ecuador, and *Orcinus orca* from California with a similarly low percentage proportion of polyunsaturated fatty acids.

Fatty acid	Surface zooplankton	*Mobula birostris*	Orca (offshore)	Orca (resident)	Orca (transient)
ΣSFA	36.6 ± 12.5	58.6 ± 11	17.1 ± 2.5	14.5 ± 2	16.7 ± 3.78
ΣSCMUFA	5.1 ± 1.9	2.1 ± 2	25.8 ± 2.7	31.8 ± 4	39.4 ± 6.4
ΣLCMUFA	13.3 ± 3	34.8 ± 11.8	49.3 ± 2.4	47 ± 4.2	39.1 ± 7.6
ΣPUFA	44.9 ± 12.6	4.5 ± 6.2	7.8 ± 1.9	6.7 ± 1.4	4.8 ± 1.8
ΣΩ3	40.9 ± 11.4	1.4 ± 4.7	4.5 ± 2	3.6 ± 1.2	2.1 ± 1
ΣΩ6	4 ± 1.3	3.1 ± 3.4	2.3 ± 0.4	2 ± 0.3	1.6 ± 0.6
Ω3:Ω6	9.7 ± 3.1	0.2 ± 0.4	1.9	1.8	1.3
Reference	This study	This study	[32]	[32]	[32]

Functional groupings of FA compositions are shown as: total saturated fatty acids (ΣSFA); total short-chain monounsaturated fatty acids (≤C16) (ΣSCMUFA); total long-chain monounsaturated fatty acids (>C16) (LCMUFA); total polyunsaturated fatty acids (ΣPUFA); total omega-3 fatty acids (ΣΩ3) and total omega-6 fatty acids (ΣΩ6).

**Table 2:** Fatty acid (FA) composition (% of total FA ± s.d.) of *Mobula birostris* muscle and whole surface zooplankton collected from Isla de la Plata, Ecuador, alongside FA profiles of *Mobula alfredi* and *Rhincodon typus* from different oceanic regions.

Fatty acid	Surface zooplankton	Surface zooplankton	*M*. *birostris*	*M*. *birostris*	*M*. *alfredi*	*M*. *alfredi*	*R*. *typus*	*R*. *typus* 2013	*R*. *typus* 2014
	Cluster 1	Cluster 2	Cluster A	Cluster B	E. Aust	Mozambique	Mozambique	W. Aust	W. Aust
14:0	7.5 ± 1.6	7 ± 3.2	1.3 ± 1.1	3.1 ± 5.4			0.6 ± 0.1	0.1 ± 0.1	0.2 ± 0.0
15:0	1.3 ± 0.2	1.2 ± 0.6	0.1 ± 0.3	0.1 ± 0.4			0.4	0.1 ± 0.0	0.2 ± 0.0
16:0	0.2±11.5	23.1 ± 8.6	26 ± 3.2	31 ± 5.9	14.7 ± 0.4	13.8 ± 0.5	12.2 ± 0.4	11.9 ± 1.4	9.7 ± 0.5
17:0	2 ± 0.4	1.6 ± 0.8	0.1 ± 0.4	0.1 ± 0.3		1.6 ± 0.1	1.5 ± 0.1	0.8 ± 0.1	1.0 ± 0.0
18:0	8.7 ± 1.2	7 ± 4.8	24.1 ± 2.2	23.9 ± 4.1	16.8 ± 0.4	17.8 ± 0.5	17.7 ± 0.3	32.0 ± 3.4	18.0 ± 0.5
20:0	0.9 ± 0.3	0.6 ± 1.1	1.1 ± 1.1	0.5 ± 1.2			0.3 ± 0	0.8 ± 0.1	0.8 ± 0.4
22:0	0.8 ± 0.2	0.5 ± 1.6	7.8 ± 5.8	0.8 ± 1.9			0.9 ± 0.1	0.8 ± 0.2	0.5 ± 0.0
23:0	0.7 ± 0.2	0.5 ± 3.6					0.6 ± 0		
**ΣSFA**	**22.5 ± 9.7**	**41.7 ± 10.5**	**60.7 ± 7.3**	**59.6 ± 7.7**	**35.1 ± 0.7**	**39.1 ± 0.7**	**37.4 ± 0.1**	**48.5 ± 3.2**	**33.2 ± 0.8**
16:1ω7	6.1 ± 0.9	4.4 ± 2.2	2 ± 1.6	1.7 ± 1.5	2.7 ± 0.3	2.1 ± 0.3	1.9 ± 0.2	0.9 ± 0.3	1.3 ± 0.1
18:1ω9t	0.3 ± 0.2	0.2 ± 7.1	17.2 ± 7.5	0.1 ± 0.4					
18:1ω9c	7 ± 1.7	5.8 ± 2.4	8.2 ± 2	22.7 ± 5	15.7 ± 0.4	16.7 ± 0.7	16 ± 0.5	13.1 ± 1.9	15.6 ± 0.7
18:1ω7	3.1 ± 0.6	2.4 ± 1.4	0	6.2 ± 3.1	6.1 ± 0.2	4.6 ± 0.5	4.3 ± 0.3	3.5 ± 0.5	4.1 ± 0.4
20:1ω9	0.7 ± 0.3	0.7 ± 0.5	0.4 ± 0.7	1.1 ± 1.5	1.0 ± 0.03	0.7 ± 0.02	0.7	1.2 ± 0.4	1.4 ± 0.2
22:1ω9	0.4 ± 0.8	1.1 ± 2	4.8 ± 4.2	4.8 ± 6.5			0.2	0.7 ± 0.2	0.6 ± 0.3
24:1ω9	1.9 ± 0.4	1.3 ± 0.6	0.2 ± 0.6	0.7 ± 1.2	1.1 ± 0.1	1.9 ± 0.1	2.3 ± 0.1	0.8 ± 0.1	1.6 ± 0.1
**ΣMUFA**	**20.2 ± 3.1**	**16.3 ± 7**	**32.9 ± 4.4**	**37.5 ± 7.6**	**29.9 ± 0.7**	**31.0 ± 0.9**	**30.2 ± 0.1**	**25.4 ± 2.6**	**30.7 ± 1.2**
18:2ω6c	1.7 ± 0.2	1.4 ± 1.5	2.4 ± 2.2	0.3 ± 0.8				0.7 ± 0.0	1.0 ± 0.1
18:3ω6	0.2 ± 0.2	0.2 ± 0.1	1.5 ± 4.4	0.1 ± 0.3					
18:3ω3	1.2 ± 0.3	1 ± 0.8		0.2 ± 0.8				0.1 ± 0.0	0.6 ± 0.3
20:4ω6 (ARA)	2.4 ± 0.4	1.6 ± 0.7	2 ± 1.8	1.9 ± 2.2	11.7 ± 0.8	16.9 ± 0.6	17.8 ± 0.4	12.5 ± 1.7	16.4 ± 1.0
20:5ω3 (EPA)	13.9 ± 3	9.4 ± 4.2			1.2 ± 0.1	1.1 ± 0.1	1.2 ± 0.1	1.7 ± 0.2	2.0 ± 0.3
22:5ω3	1.8 ± 1.3	1.7 ± 1	0.2 ± 0.5		2.0 ± 0.1	2.1 ± 0.1	2.5 ± 0.1	0.1 ± 0.0	0.1 ± 0.0
22:6ω3 (DHA)	34.6 ± 4.9	25.9 ± 9.7	0.1 ± 0.4	0.3 ± 0.8	10.0 ± 0.5	2.5 ± 0.2	2.8 ± 0.2	2.4 ± 0.2	3.5 ± 0.3
**ΣPUFA**	**57.3 ± 8**	**42 ± 15.2**	**6.3 ± 5**	**2.9 ± 3.1**	**34.9 ± 1.2**	**29.9 ± 0.9**	**32.4 ± 0.1**	**26.1 ± 2.9**	**36.2 ± 0.9**
ΣΩ3	52.1 ± 7.5	38.3 ± 15	0.3 ± 1	0.6 ± 1.3	13.4 ± 0.6	6.1 ± 0.3	6.5 ± 0.1		
ΣΩ6	5.1 ± 0.7	3.6 ± 1.9	5.9 ± 4.8	2.3 ± 2.4	21.0 ± 1.4	23.8 ± 0.8	25.9 ± 0.1		
Ω3/Ω6	10.4 ± 1.6	10.7 ± 4.8	0.1 ± 0.2	0.1 ± 0.3	0.7	0.3	0.3	0.3 ± 0.1	0.5 ± 0.1
Others*	2.2 ± 0.1	1.4 ± 0.1	0.6 ± 0.1	0.4 ± 0.1					
18:1ω7/18:1ω9	0.4	0.9	0.03	0.3	0.4	0.3		0.3	0.3
EPA/DHA	0.4	0.4	0.1	0	0.1	0.4		0.7	0.6
16:1ω7/16:0	30.5	0.2	0.1	0.1	0.2	0.2		0.1	0.1
Reference	This study	This study	This study	This study	[13]	[13]	[33]	[14]	[14]

Clusters A and B, and clusters 1 and 2 designate distinct FA profiles groups of *M*. *birostris* and surface zooplankton, respectively, as revealed by cluster analysis. Also included are the FA compositions of *Mobula alfredi* from eastern Australia (± s.e.) (E. Aust) and Mozambique [13], and *Rhincodon typus* from Mozambique (± s.e.) [33] and western Australia (W. Aust) (± s.e.) [14]. Functional groupings of FA compositions are shown as: total saturated fatty acids (ΣSFA); total monounsaturated fatty acids (ΣMUFA); total polyunsaturated fatty acids (ΣPUFA); total omega-3 fatty acids (ΣΩ3); total omega-6 fatty acids (ΣΩ6); Arachidonic acid (ARA); Eicosapentaenoic acid (EPA); docosahexaenoic acid (DHA).

*Fatty acids comprising <1% of FA profile; 18:2ω6 trans, 22:0, 15:1, 14:1ω5, 19:0, 24:0, 17:1, 20:1ω7, 18:4ω3, 20:3ω6, 20:3ω3, 22:4ω6, 20:2ω6.

At 70% similarity, cluster analysis revealed two distinct FA profile groups (A and B) for *M*. *birostris* (Fig 1), with significant differences between the FA profiles of individuals from each group (ANOSIM, *R* value = 0.9062, p < 0.001) (Table 2). There were low levels of PUFA detected in both *M*. *birostris* clusters, but of the PUFAs present, ω6 dominated. Cluster A and B both had ω3/ω6 ratios of 0.1 (Table 2). Cluster A comprised of male and female samples from 2014, whereas Cluster B comprised males and females across all sampling years (2012–2014).

The overall similarity between *M*. *birostris* cluster A and B was 64.1% (SIMPER) and major differences between A and B were due to variations in 18:1ω9 isomers (Table 2, S3 Table). Cluster A had higher mean percentage of 18:1ω9t, whereas 18:1ω9c was higher in cluster B (Table 2). Other FAs that contributed to major differences between *M*. *birostris* cluster groups were 22:0 (10.3%), 16:0 (9.1%), 18:1ω7 (8.9%), 22:1ω9 (7.5%) and 18:0 (5%) (S3 Table).

### Surface zooplankton fatty acid profile

The FA profiles of surface zooplankton collected during ‘feeding’ and ‘non-feeding’ events at Isla de la Plata did not significantly differ (ANOSIM, *R* value  =  0.0069, p = 0.43). There was no significant difference in the FA profiles of zooplankton sampled among different years (ANOSIM, *R* value = 0.0348, p = 0.25) or collection sites (ANOSIM, *R* value  =  0.0772, p = 0.18), while there was a significant difference among zooplankton collected in different months (ANOSIM, *R* value = 0.2586, p <0.05) and temperatures (ANOSIM, *R* value = 0.163, p <0.05) (S1 Table). For all years, pairwise comparisons revealed a significant difference between August and September, and September and October (p <0.05), but no significant differences between August and October (p = 0.14). However, upon ordination, no discrete clusters were formed and there was a high degree of overlap between each month (S1 Fig). For all temperature groupings (20, 21, 22, 23 and 24°C), pairwise comparisons revealed only one significant difference, which was between the FA profiles of surface zooplankton collected in 20°C and of those collected in 24°C (p <0.05), with samples collected in 20°C generally characterised by having lower proportions of 16:0 and PUFA, compared to samples collected in 24°C (S1 Table). However, upon ordination, there was large overlaps between each temperature grouping (S2 Fig).

**Fig 3:**
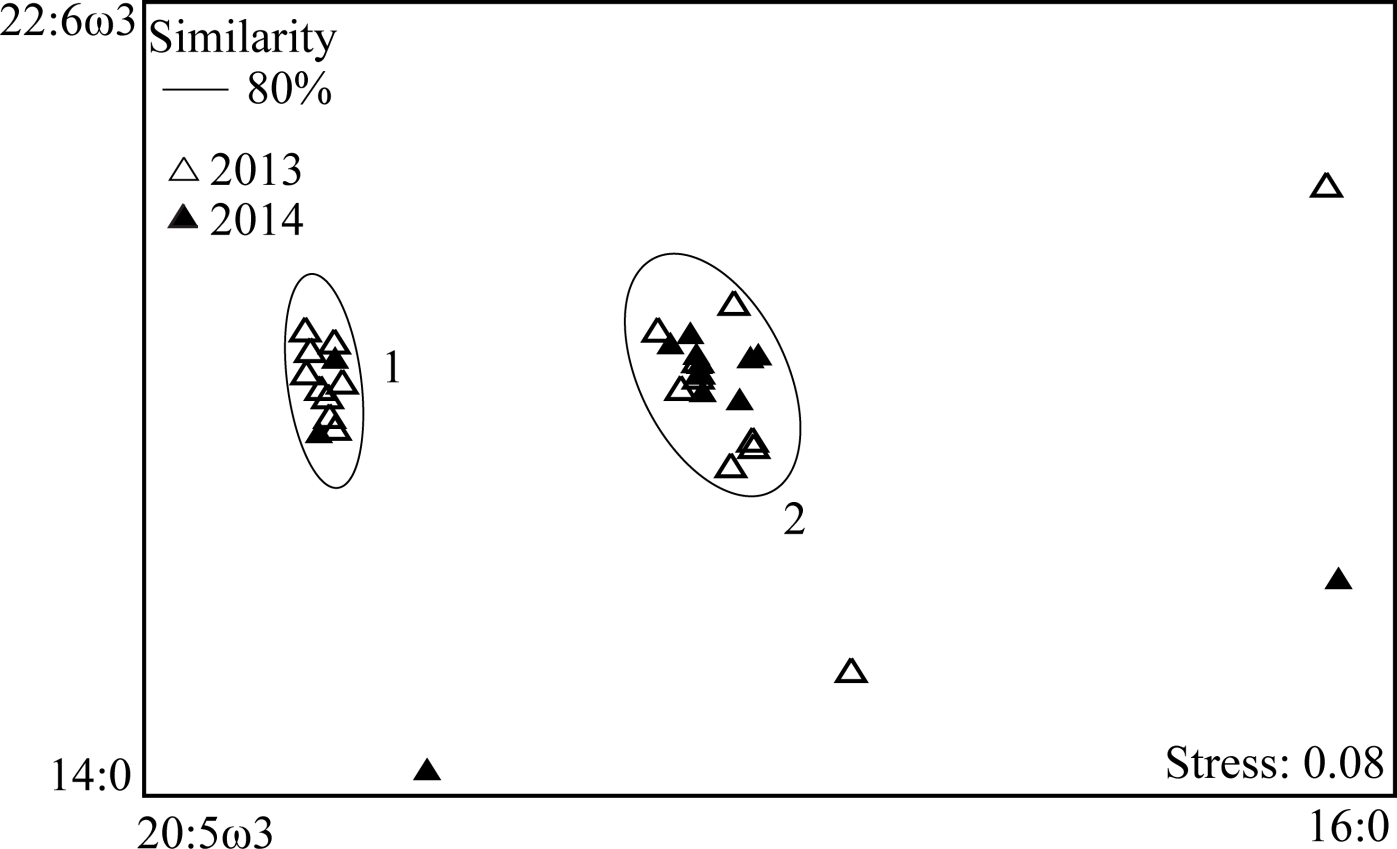
Non-metric multi-dimensional scaling plot for surface zooplankton fatty acid profiles (Bray-Curtis Similarity Index). Fatty acid labels represent the main coefficients (>0.7) contributing to each axis. Cluster 1 and 2 designate the two distinct FA profile groups of surface zooplankton as revealed by cluster analysis at 80% similarity.

Overall, surface zooplankton from Isla de la Plata predominantly comprised PUFA (44.9 ± 12.6%) (Table 2). Cluster analysis at 80% similarity revealed there to be 2 discrete clusters (1 & 2) of surface zooplankton collected and 4 outlier samples (Fig 3, S4 Table).

The major FA for both zooplankton clusters was DHA followed by EPA for cluster 1 and 16:0 for cluster 2 (Table 2). Both surface zooplankton clusters had similar levels of ARA to *M*. *birostris* and clusters 1 and 2 had mean ω3/ω6 ratios of 10.4 and 10.7, respectively (Table 2). While both cluster 1 and 2 were predominantly comprised of calanoid copepods, cluster 1 samples contained a slightly higher abundance of chaetognaths in comparison to cluster 2. The EPA/DHA ratio for both surface zooplankton groups was 0.4. The FA profiles of surface zooplankton collected from Isla de la Plata were dominated by DHA and had a high ω3/ω6 ratio (11.8) in comparison to *M*. *birostris* (0.6).

### Lipid class profiles

*Mobula birostris* LC profiles were dominated by PL (82.9 ± 6.3%), followed by ST (9.5 ± 5%), and FFA (4.3 ± 3.4%) (Table 3). Lipids from surface zooplankton were dominated by TAG/WE (38.5 ± 13.9%) followed by PL (30.1 ± 9.1%) and then FFA (19.3 ± 16.2%) (Table 4).

**Table 3:** Lipid class (LC) profiles, reported as % of total lipid, for *Mobula birostris* from Ecuador (this study), alongside *Mobula alfredi* from Mozambique and eastern Australia [13], and *Rhincodon typus* from western Australia [14] and Mozambique [33].

Lipid Class	*M*. *birostris*	*M*. *alfredi*	*M*. *alfredi*	*R*. *typus*	*R*. *typus*
	Ecuador (n = 10)	E. Australia (n = 14)	Mozambique (n = 6)	W. Australia (n = 52)	Mozambique (n = 24)
TAG/WE	3.7 ± 2.2	4.5	5.1	11.4	5.1
FFA	4.3 ± 3.4	2.4 ± 0.5	3.0 ± 0.4	2.0 ± 0.6	5.4 ± 0.7
ST	9.5 ± 5	8.0 ± 0.7	13.4 ± 2.2	14.6 ± 1.3	21.4 ± 0.7
PL	82.9 ± 6.3	85.2 ± 0.8	78.5 ± 2.5	71.9 ± 3.0	68.1 ± 2.2
Reference	This study	[13]	[13]	[14]	[33]

Lipid classes shown; wax esters (WE), triacylglycerols (TAG), free fatty acids (FFA), sterols (ST) and phospholipids (PL).

**Table 4:** Average lipid class (LC) profiles of surface zooplankton from a *Mobula birostris* aggregation site in Ecuador (this study), and from *Mobula alfredi* aggregation sites in eastern Australia and Mozambique [13].

Lipid Class	Surface Zooplankton		
	Ecuador (n = 6)	E. Australia (n = 38)	Mozambique (n = 8)
TAG/WE	38.5 ± 13.9	24	6.2
FFA	19.3 ± 16.2	17.1 ± 0.9	57.2 ± 2.1
ST	7.5 ± 7.3	5.5 ± 0.2	6.4 ± 0.5
PL	30.1 ± 9.1	53.5 ± 2.1	30.2 ± 1.5
Reference	This study	[13]	[13]

Reported as % of total lipid with lipid classes shown; wax esters (WE), triacylglycerols (TAG), free fatty acids (FFA), sterols (ST) and phospholipids (PL).

## Discussion

The FA profiles of *Mobula birostris* and surface zooplankton were markedly different apart from similar proportions of ARA, which suggests a small reliance on surface zooplankton for dietary intake in *M*. *birostris*. This complements previous stable isotope data that estimated surface zooplankton sources on average contributed a small portion of dietary intake (27%) in comparison to mesopelagic sources (73%) [15]. The principal PUFA for *M*. *birostris* was ARA, which is also one of the main PUFA for *Mobula alfredi* and *Rhincodon typus* from Australia and Mozambique [12, 14], which suggests a common dietary source between these species. However, the proportion of ARA was much lower in the FA profile of *M*. *birostris* from the eastern Pacific than in FA profiles of planktivorous elasmobranchs from other study regions, and was instead, more similar to the proportions of ARA found in the muscle tissue of deep-sea chondrichthyans species including the South China catshark *Apristurus sinensis*, Shortnose spurdog *Squalus megalops* and Southern chimaera *Chimaera fulva* [34].

### PUFA-poor fatty acid profiles of giant manta rays

The relative lack of PUFA in the FA profile of *M*. *birostris* is an atypical and novel finding among all planktivorous elasmobranch fatty acid studies to date. This could be attributed to degradation of *M*. *birostris* samples, however, this was deemed minimal as average FFA values were <5% and the muscle contained high proportions of PL. In comparison, *M*. *alfredi* had similar FFA amounts (2.4–3%), but had FA profiles dominated by PUFAs [13]. A lack of PUFAs in the FA profile of *M*. *birostris* muscle could indicate their long-term diet comprises an uncharacterised, ‘PUFA-poor’ food source and that there is an absent capacity for endogenous biosynthesis of long-chain PUFA in this species. Alternatively, muscle tissue in elasmobranchs is relatively lipid poor in comparison to the liver [35]. Here, the ‘PUFA-poor’ FA profile of *M*. *birostris* muscle could be representative of preferential breakdown of PUFA and/or designated routing of PUFA to the liver. In addition, the ‘PUFA-poor’ FA profile of *M*. *birostris* could also be a function of the geographic zone that this species inhabits [36] due to temperature, light and nutrient availability affecting FA patterns in the marine environment [18]. Typically, in baseline algal communities, proportions of unsaturated FAs increase in cooler temperatures [37], omega-3 PUFAS increase under non-limiting light conditions and during exponential growth phases [38, 39], and omega-6 PUFAS increase under decreased light intensity [38]. Therefore, the ‘PUFA-poor’ profile of *M*. *birostris* may be a result of inhabiting cooler waters (at depth or lower latitudes) and feeding predominantly during decreased light intensity (at depth or at night).

In stable isotope studies, elasmobranch muscle tissue can take over a year to reflect an ingested prey item [27], and biopsied muscle is only representative of ecological interactions at the sampling site if the study organism has fed there for this entire period of time. The occurrence of *M*. *birostris* off mainland Ecuador is highly seasonal and biopsied muscle therefore is likely to be representative of *M*. *birostris* feeding activity within a PUFA-poor environment before arrival to the aggregation site in Ecuador. Presuming surface zooplankton at Isla de la Plata are representative of surface zooplankton in the surrounding region throughout the year, surface waters in the eastern equatorial Pacific are seemingly not PUFA- poor environments. Although this is a broad assumption, surface zooplankton FA profiles off mainland Ecuador were not markedly different to FA profiles for surface zooplankton collected throughout the year from another tropical oceanic region, The Great Barrier Reef (eastern Australia) or to FA profiles for surface zooplankton collected from southern Mozambique in austral summer [13]. Surface zooplankton FA profiles from all three of these regions were dominated by omega-3 FAs, in the order of DHA, EPA and ARA. Omega-3-to omega-6 ratios were also similar among these regions, with Mozambique having a slightly higher ratio (12.8) compared to eastern Australia (9.5) and Ecuador (10.4).

Variations in essential fatty acid requirements for different fish species reflects different dietary and metabolic adaptations to different habitats (Sargent. Et al 1999). There is no information on how manta rays allocate FA in their tissue, although previous studies show that muscle from the reef manta ray in eastern Australia and Mozambique has a relatively standard composition [13]. The inner blubber of killer whales from the NE Pacific also contained low proportions of PUFA (~4.8–7.8% of total FA) and high proportions of MUFA [32]. Blubber FA compositions are likely to be altered relative to ingested FAs due to selective metabolism of certain FAs prior to their disposition in the blubber [40]. However, the FA profile of inner blubber from Arctic Bowhead whales and Mediterranean Fin whales all contained >20% PUFA [41, 42]. Killer whales and *M*. *birostris* are the only two large migratory vertebrate species with published FA profiles in the temperate to tropical eastern Pacific, and there are no FA profiles for killer whales or *M*. *birostris* outside of this region. Therefore, it is difficult to assign the lack of PUFA seen in these two species as species specific, or a metabolic adaptation to this particular region [36]. Interestingly, muscle tissue from obligate hydrothermal vent tonguefishes from the western Pacific (*Symphurus* spp.) contained relatively high proportions of PUFA (mean 41.6%) [43], in comparison to the obligate hydrothermal vent zoarcid fish *Thermarces cerberus* from the eastern Pacific (East Pacific Rise, ~3000 km NW from Isla de la Plata), which was characterised by lower relative amounts of PUFA (mean 18.4%) [44]. This indicates that there could be a gradient of proportions of PUFA in the FA profiles of fishes across the Pacific Ocean, with higher proportions found in the west than the east, however, additional sampling of fish species that are found in both the west and east Pacific would be needed to confirm this hypothesis. Further sampling of prey baselines from the tropical eastern Pacific at different depths and during night time hours, where surface assemblages change dramatically due to diurnal vertical migrating species [45], is also required to establish the origin of the PUFA-poor food source reflected in the FA profile of *M*. *birostris*.

Mesopelagic copepods (0 – 300m) from the Costa Rica Dome contain low to moderate proportions of PUFA (3.5–22% total FA), with one particular species, *Rhinocalunus rostifrons* shown to have on average 5% PUFA over two collection years. This is in contrast to mesopelagic zooplankton from Hawaii (0–1000 m), which contained high proportions of PUFA [46]. There are no published mesopelagic zooplankton FA profiles from the suspected home range of *M*. *birostris* in the eastern equatorial Pacific, which comprises mainland Ecuador, the Galapagos Islands and northern Peru [17] and unfortunately, due to logistical constraints, we could not sample mesopelagic zooplankton from the region. The dominant zooplankton in the Peru upwelling region is *Euphasia macronata* [47], which during the day is typically found at mesopelagic depths ~300 m, with daytime net tows completely devoid of this species [48, 49]. However, at night *E*. *macronata* undertakes large vertical migrations and can be found feeding near surface waters at ~15 m [49]. Euphausiids are the dominant prey type for other planktivorous mobulid rays: *Mobula thurstoni* and *M*. *japanica* from the Gulf of California [50], and *M*. *thurstoni* from the Western Atlantic [51]. They are also a common prey item for other planktivorous species including baleen whales [52], whale sharks *Rhincodon typus* [53] and megamouth sharks *Megachasma pelagios* [54]. Given the ubiquity of krill- feeding by large planktivores and the large abundance of *E*. *macronata* in close proximity to Isla de la Plata, it is possible that this mesopelagic zooplankton is a major prey source for *M*. *birostris*. However, a caveat still remains in that if *E*. *macronata* migrate to surface waters to feed, these surface waters during the day are not PUFA-poor environments. Further sampling and the determination of FA profiles of night-time surface zooplankton assemblages over continental shelf habitat where large abundances of *E*. *macronata* reside during the day are required to resolve whether this krill species is a major dietary item for *M*. *birostris*.

### Dietary derived fatty acids in giant manta rays

Arachidonic acid (ARA) was the PUFA with the highest proportion in the FA profile of *M*. *birostris*, but still comprised very little of the total FA profile (~2%). In fishes, DHA and EPA, but not ARA, are typically the major PUFAs of cell membranes [55]. Although ARA is thought of as a minor component in fish cell membranes, its role has been largely unassessed. Here, ARA was the dominant PUFA in the profile of *M*. *birostris*, with only trace amounts of DHA found and no EPA. ARA is a major precursor of eicosanoids in fishes, which are produced in response to stressful situations at both a cellular and body level [56]. EPA is also a major precursor to eicosanoids, albeit less biologically active ones than those formed from ARA. EPA competitively inhibits the formation and actions of eicosanoids from ARA, therefore, high tissue ratios of ARA: EPA result in enhanced eicosanoid actions [55]. Higher proportions of ARA than EPA seen here could be linked to an environmentally induced stressor. For example, low food availability or changes in dissolved oxygen concentrations throughout the water column in the eastern tropical Pacific; a region of near constant hypoxia in surface waters and with suboxic conditions at depths of 300–500 m [55, 57, 58]. Alternatively, FA profiles are species-specific [59], and given there are no published FA profiles for *M*. *birostris* from other regions, the lack of PUFA might be a consequence of species-specific energy storage and cell mechanisms as opposed to being a result of conditions in the eastern equatorial Pacific.

Typically, photosynthetic organisms are the only organisms that can biosynthesize 18:2ω6 and 18:3ω3 *de novo* [18], with the derivatives from these PUFAs (ARA, EPA and DHA) deemed essential constituents in heterotrophic organisms. The presence of these derivatives in higher quantities in planktivorous elasmobranchs from eastern and western Australia, and southern Mozambique indicate a higher reliance on surface food sources [6]. At *M*. *alfredi* aggregation sites in eastern Australia, surface foraging activity is commonly observed all year round [8]. In comparison, *M*. *birostris* were only observed feeding on 4% of encounters during peak aggregation time off mainland Ecuador (S2 Appendix), and likely get the majority of their diet from offshore mesopelagic sources [15].

### Other fatty acids potentially derived from diet

Essential fatty acids are defined as those that cannot be biosynthesised in sufficient quantities for normal physiological function. At present, only omega-3 and omega-6 FAs are considered essential FAs in sharks and rays. Low PUFAs here, across all *M*. *birostris* individuals sampled over multiple years and months suggests that FAs other than PUFAs are acting in an ‘essential’ capacity. Although not explicitly considered dietary FAs, the MUFA profile of *M*. *birostris* was dominated by 18:1ω9 isomers. Deep-sea organisms are richer in C18:1 FAs compared to those from shallow waters and in the liver oils of deep-sea sharks, PUFAs constitute only minor components (1–13%) [60]. Specifically, oleic acid (18:1ω9c) increases with depth and is common in bathypelagic crustaceans and fishes [61], along with upper mesopelagic copepods (0–300 m) [62] and upper and lower mesopelagic (500–1000 m) zooplankton [46]. Additionally, the most common mesopelagic zooplankton off neighbouring Peru, *E*. *macronata*, is known to produce major amounts of C18 fatty acids, with oleic acid being the most abundant [47]. In deep sea sharks, the FA composition in all tissues is dominated by 18:1ω9, with content of this FA reported at 21–43% [63]. As well as energy storage, it has also been proposed that high proportions of C18:1 FAs might be an adaptive response to high pressure in deep waters [64]. Satellite tagging of *M*. *alfredi* and closely related *Mobula tarapacana* have shown that these predominantly surface dwelling rays can dive to depths of 432 m and 2000 m, respectively [65, 66]. High proportions of oleic acid in *M*. *birostris* muscle tissue are likely mesopelagic in origin, however, another important consideration is that vertically migrating mesopelagic zooplankton, such as euphausiids and myctophid fishes, are hugely abundant [67, 68]. These species undertake extensive diel vertical migrations from below 200–1000 m to surface waters during night time hours to feed [49, 69], and comprise and important food source for *M*. *birostris* in the Philippines [70]. Complementary electronic tagging studies are needed to validate whether this *M*. *birostris* is targeting mesopelagic prey in shallow or deeper waters, or a combination of both.

Overall, the MUFA profile of *M*. *birostris* contained high amounts of 18:1ω9 isomers, with proportions of the trans-isomer (elaidic acid) higher in Group A in comparison to group B where the cis-isomer (oleic acid) dominated. In killer whales, FA profiles can distinguish between ‘transient’ ‘offshore’ and ‘resident’ populations (Herman et al., 2008), and in bowhead whales the variability of FA profiles within populations has been linked with differences in phytoplankton-derived FA [42]. Cis- and trans-isomers often have different physical properties and in this case, elaidic acid has a more symmetrical shape. This symmetry enables a larger number of trans-molecules to pack tightly into a space in comparison to the unsymmetrical cis- molecules. The increase in elaidic acid composition could therefore be linked with a more efficient energy storage mechanism in the subset of individuals from 2014 (Group A) and demonstrative of these individuals occupying more nutrient depleted offshore environment.

### Lipid content and class composition

Lipid content (LC) profiles of *M*. *birostris* muscle contained low proportions of triacylglycerols (TAG). In chondrichthyan species, TAG is usually stored in the liver [71] and muscle used here may be an inappropriate proxy for energy storage capabilities. The LC profile of *M*. *birostris* from the eastern Pacific was dominated by phospholipids (PL) and comparable to the LC profiles of *M*. *alfredi* from eastern Australia and Mozambique [13]. The observed LC profiles are comparable to those of deep water and demersal sharks, which have relatively high levels of structural components (PL) and low or zero of energy storage lipids (TAG/WE) [34, 71].

Some zooplankton samples had relatively high amounts of FFAs. However, the same samples all contained high proportions of PUFA, indicating that if there was degradation, this was solely restricted to lipid class composition [72]. Surface zooplankton from Isla de la Plata, Ecuador had similar levels of FFA and ST to surface zooplankton from eastern Australia [13]. However, these east and west Pacific sampling sites differed in their primary constituents with TAG/WE being dominant in the east Pacific (this study), while PL were the dominant lipid class constituent in the W. Pacific [13]. TAG is usually associated with energy storage in marine organisms [73], and it is possible that this is an adaptation of zooplankton in the eastern Pacific which is as a region is subject to El Niño–Southern Oscillation mediated weather events, and subsequent fluctuations in productivity [74]. Further sampling of surface zooplankton from more sites in the tropical eastern and western Pacific would be needed to more comprehensively investigate these difference in LC profiles.

### Limitations of molecular tracers to infer diet

The allure of non-lethal and cost effective techniques often means molecular approaches are being more readily used to reconstruct diets for migratory and threatened species. However, the robust applicability of FA analysis in higher trophic level organisms is highly dependent on prior knowledge of how FA profiles are altered through *de novo* biosynthesis, metabolisation and breakdown of dietary FAs. These processes are regulated by life history strategies, the environment and type of lipid storage [18]. Molecular approaches are most robust when there is *a priori* knowledge on diet through more traditional approaches such as stomach contents analysis [75].

In the case of elusive and threatened species, stomach contents data and faeces are often unavailable, and prior knowledge of feeding ecology is instead based on observational accounts of foraging activity. When migratory species feed away from sight, on a variety of different prey, and in areas with poorly characterised molecular baseline values, it is difficult to make robust inferences on diet from molecular profiles alone. The interpretation of FA analyses remains hindered by our limited knowledge of lipid metabolism and FA biosynthesis in elasmobranchs, along with unknown turnover times of different tissues. However, certain scenarios can be discounted given that FA profiles of prey are assimilated relatively intact into consumer elasmobranch muscle tissue [76]. This study is an example of such a scenario, where the suspected surface zooplankton prey source was PUFA rich and theoretically, these PUFA would be assimilated and apparent in the *M*. *birostris* consumer if surface zooplankton collected during the day comprised a substantial portion of their diet. Ultimately, to support the study of wild populations, aquaria studies would be needed to investigate *de novo* biosynthesis, metabolisation and breakdown of dietary FAs in planktivorous elasmobranchs.

### Conclusions

Better management of fishing activity requires information on areas where foraging activity and fisheries overlap [77] and the combination of results from dietary molecular analyses with movement and fisheries data will enable more robust estimations on habitat and prey preferences of vulnerable elasmobranch species [78]. *Mobula birostris* is a species that has the ability to move into deep and physiologically inhospitable environments [79], and their molecular profile (stable isotopes and fatty acids) indicates this vertical habitat use adaptation could be crucial for long-term dietary intake [15]. In the nearby Galapagos region, climate change is predicted to reduce the amount of available deep sea habitat for yellow fin tuna [80], and a potential reduction of usable vertical habitat in this region for *M*. *birostris* could negatively impact a species that already has little capacity to withstand directed fishery pressure [81].

## Supporting information

S1 Appendix Detailed methodology for lipid extraction and signature fatty acid analysis. (DOCX)

S1 File Individual *Mobula birostris* and surface zooplankton fatty acid profiles, along with sampling date and location. (XLSX)

S1 Fig Comparison of surface zooplankton signature fatty acid (FA) profiles among sampling months with 95% ellipses. Non-metric multi-dimensional scaling ordination of surface zooplankton FA profiles sampled at Isla de la Plata, Ecuador, from August 2013 to October 2013, and August 2014 to September 2014. There was a significant difference among samples (ANOSIM, R value = 0.2586, p <0.05), with pairwise comparisons revealing a significant difference between August and September, and September and October (p <0.05), but no significant differences between August and October (p = 0.14).
(EPS)

S2 Fig Comparison of surface zooplankton signature fatty acid (FA) profiles between different *in situ* temperature groupings at time of collection. Non-metric multi-dimensional scaling ordination of surface zooplankton FA profiles sampled at Isla de la Plata, Ecuador, from August—October 2013, and August—September 2014. There was a significant difference in the fatty acid profile of surface zooplankton between temperature groupings (ANOSIM, R value = 0.163, p < 0.05), with pairwise comparisons revealing a significant difference between 20°C and 24°C (SIMPER, p < 0.05).
(EPS)

S1 Table Signature fatty acid (FA) profiles (% of total FA ± s.d.) of surface zooplankton collected from Isla de la Plata, Ecuador among sampling months and temperature groupings. Here, the FA profiles of surface zooplankton were significantly different among sampling months and temperature groupings (SIMPER, p <0.05).
(DOCX)

S2 Table Signature fatty acid (FA) profiles (% of total FA ± s.d.) of *Mobula birostris* among sample collection years. The FA profiles of *M*. *birostris* were not significantly different among years (ANOSIM, R = 0.04753, p = 0.27).
(PDF)

S3 Table Similarity percentage analysis (SIMPER) results of *Mobula birostris* fatty acid profiles for cluster groups A and B (70% similarity). Fatty acids with an average contribution >5% are included and data was not transformed prior to analysis.
(PDF)

S4 Table Similarity percentage analysis (SIMPER) of surface zooplankton fatty acid profiles among cluster groups 1 and 2 (80% similarity). Fatty acids with an average contribution >5% are included and data was not transformed prior to analysis.
(PDF)
