# Supplementary material for: Novel signature fatty acid profile of the giant manta ray suggests reliance on an uncharacterised mesopelagic food source low in polyunsaturated fatty acids
Source: PLoS One. 2018 Jan 12;13(1):e0186464. doi: 10.1371/journal.pone.0186464 (PMC5766321; doi:10.1371/journal.pone.0186464)
Supplement: S1 Appendix — (DOCX) [file pone.0186464.s001.docx]

**S1 Appendix**

*Lipid Extraction*

For the one-day method, 10 – 40 mg of freeze dried *Mobula birostris* muscle was weighed out then 0.24 mL distilled water and 6 mL of 2:1 chloroform:methanol (2:1) was added. The mixture was stirred for ~1 minute, left to rest for 10 minutes, and then stirred again. The mixture was then poured into a syringe with a GF/C filter paper at the bottom and was filtered into a centrifuge tube. The tube was washed by the addition of 6 mL chloroform:methanol (2:1) solution and remixed for 30 seconds. This wash solution was then poured again through the syringe and filter paper. 3 mL of 0.9 % NaCl was added to the centrifuge tube and contents were thoroughly mixed through inversion and centrifuged (5 min, 2500 rpm). The top layer was then discarded and 2.2 mL 50 % MeOH was added to each sample. Contents were again mixed by inversion and centrifuged (5 min, 2500 rpm). The top layer was discarded again and the mixture was filtered through Na2SO4 filter (20 mL syringe, 3 mL Na2SO4) into a 25 mL cylinder. The screw cap centrifuge tube and the syringe were washed with a small amount of pure chloroform to remove any residual lipids.

For the three-day (Folch et al., 1957) method, a homogenized wet *M. birostris* sample (15 – 100 mg) was weighed out and 10 mL of chloroform:methanol (2:1) was added. The contents were mixed and left to settle for 1 hour. Contents were then filtered into a separating funnel ensuring the removal of all solids. Another 10 mL of chloroform:methanol (2:1) was added to the filtered sample to wash any residual lipids into the separating funnel. 5 mL of 0.9 % NaCl was then added to the solvent and the funnel was closed and shaken to mix the NaCl and solvent solution. The funnel was then left at 4 °C for 6 hours to separate into water and solvent layers. The lower solvent phase was drained off and the upper layer was discarded. The lower solvent phase was added to the separating funnel again and 5 mL of 1 % NaCl:Methanol (1:1) was added. The funnel was closed and shaken again to mix the contents. The funnel was then left at 4 °C for 12 hours to separate into water and solvent layers. The lower phase was again drained off into an evaporating flask and the upper layer was discarded.

Non-lipid material was discarded and the total lipid extract (LE) from both methods was dried under a stream of inert nitrogen gas and weighed, and was then stored at −18 °C.

*Fatty acid signature analysis*

Fatty acid methyl esters (FAME) were obtained by transferring the LE into a pyrex screw cap test tube, this was then dried under a stream of inert nitrogen gas. To the dried LE, 1.0ml of MeOH:Toluene (3:2) and 1.0 mL of Acetyl chloride: methanol (1:20) (freshly prepared on ice) was added. The tube was then flushed with nitrogen and the stopper was tightly replaced and shaken. Test tubes were then placed on a heating block at 100 °C for 1 hour. Tubes were allowed to cool, then 0.3 mg of internal standards methyl heneicosanoic acid (Me C21:0) and tricosanoic acid (C23:0) were added. 1.5 mL hexane and 1 mL water were then washed into the tube and the tube was flushed with nitrogen and shaken. Tubes were centrifuged at 1500 rpm for 2 min and the upper layer was pipetted off and filtered through Na_2_SO_4_ into a 4.5 mL HPLC vial. The hexane extraction was then repeated 2 more times and the collected upper layers were mixed and collected into a 1.5 mL vial for gas chromatography (GC) analysis.
