# Supplementary material for: Novel signature fatty acid profile of the giant manta ray suggests reliance on an uncharacterised mesopelagic food source low in polyunsaturated fatty acids
Source: PLoS One. 2018 Jan 12;13(1):e0186464. doi: 10.1371/journal.pone.0186464 (PMC5766321; doi:10.1371/journal.pone.0186464)
Supplement: S1 Table — Here, the FA profiles of surface zooplankton were significantly different among sampling months and temperature groupings (SIMPER, p <0.05). (DOCX) [file pone.0186464.s005.docx]

| Fatty Acid | Zooplankton | Zooplankton | Zooplankton | Zooplankton | Zooplankton | Zooplankton | Zooplankton | Zooplankton |
| --- | --- | --- | --- | --- | --- | --- | --- | --- |
|  | August  (n=8) | September (n=16) | October (n=8) | 20 °C  (n=5) | 21 °C  (n=2) | 22 °C  (n=3) | 23 °C  (n=10) | 24 °C  (n=12) |
| 14:0 | 8.7 ± 2.2 | 7.6 ± 2.4 | 6.8 ± 1.4 | 10.7 ± 1.5 | 11 ± 5 | 6.1 ± 1.3 | 6.8 ± 1.1 | 6.9 ± 1.1 |
| 15:0 | 1.2 ± 0.3 | 1.2 ± 0.4 | 1.1 ± 0.5 | 1.3 ± 0.3 | 0.5 ± 0.6 | 1.1 ± 0.1 | 1.3 ± 0.3 | 1.2 ± 0.4 |
| 16:0 | 10.8 ± 11.4 | 21.4 ± 9.7 | 8.4 ± 15.5 | 8.7 ± 11.8 | 32.1 ± 14.2 | 7.2 ± 12.3 | 11.6 ± 12.2 | 20.9 ± 10.8 |
| 17:0 | 1.7 ± 0.4 | 1.5 ± 0.5 | 2 ± 0.8 | 1.6 ± 0.6 | 0.6 ± 0.9 | 1.8 ± 0.4 | 2 ± 0.3 | 1.6 ± 0.6 |
| 18:0 | 7.8 ± 2.1 | 7.8 ± 1.9 | 9.9 ± 2.9 | 8 ± 2.6 | 10.5 ± 5.4 | 8.3 ± 1.4 | 7.8 ± 1.2 | 8.6 ± 2.8 |
| 19:0 | 0.2 ± 0.2 | 0.2 ± 0.1 | 0.4 ± 0.2 | 0.2 ± 0.1 | 0.1 ± 0.1 | 0.3 ± 0.1 | 0.3 ± 0.2 | 0.3 ± 0.1 |
| 20:0 | 0.6 ± 0.2 | 0.8 ± 1 | 1 ± 0.5 | 0.5 ± 0.2 | 0.2 ± 0.3 | 0.8 ± 0.4 | 0.9 ± 0.4 | 0.9 ± 1.2 |
| 22:0 | 0.5 ± 0.3 | 0.8 ± 1 | 0.7 ± 0.3 | 0.6 ± 0.2 | 0.2 ± 0.3 | 0.7 ± 0.3 | 0.6 ± 0.3 | 0.9 ± 1.2 |
| ΣSFA | 32 ± 8.9 | 41.8 ± 10.7 | 30.9 ± 15.8 | 32.1 ± 8.7 | 55.5 ± 21.9 | 27.1 ± 10 | 31.9 ± 10.3 | 41.7 ± 10.9 |
| 16:1ω7 | 6.2 ± 1.9 | 4.7 ± 1.7 | 5 ± 2.1 | 7.6 ± 1.1 | 6.2 ± 3.7 | 4.8 ± 1.2 | 5.2 ± 1.3 | 4 ± 1.5 |
| 18:1ω9c | 6.9 ± 1.9 | 5.9 ± 1.4 | 8 ± 2.5 | 7.1 ± 2.3 | 7.7 ± 0.4 | 5.5 ± 1.4 | 5.9 ± 1.2 | 7.3 ± 2.4 |
| 18:1ω7 | 3.3 ± 1.1 | 2.4 ± 0.6 | 2.6 ± 1.1 | 3.7 ± 1 | 3.2 ± 0.2 | 3 ± 0.5 | 2.6 ± 0.6 | 2.1 ± 0.8 |
| 20:1ω9 | 0.9 ± 0.3 | 0.6 ± 0.3 | 0.6 ± 0.4 | 0.7 ± 0.4 | 0.7 ± 1 | 0.6 ± 0.2 | 0.6 ± 0.1 | 0.7 ± 0.4 |
| 22:1ω9 | 0.9 ± 0.9 | 1.4 ± 1.3 | 1.4 ± 2.6 | 0.4 ± 0.4 | 3.1 ± 2.6 | 0.7 ± 0.2 | 0.9 ± 0.9 | 1.8 ± 2.2 |
| 24:1ω9 | 1.4 ± 0.4 | 1.3 ± 0.4 | 1.8 ± 0.9 | 1.4 ± 0.5 | 0.5 ± 0.8 | 2 ± 0.7 | 1.6 ± 0.5 | 1.3 ± 0.5 |
| ΣMUFA | 20.1 ± 4.8 | 16.8 ± 3.3 | 20.1 ± 1.7 | 21.8 ± 5.2 | 21.8 ± 3.9 | 17.1 ± 3.3 | 17.4 ± 3.2 | 17.7 ± 2.8 |
| 18:2ω6 cis | 1.6 ± 0.4 | 1.5 ± 0.6 | 1.3 ± 0.6 | 1.8 ± 0.4 | 0.7 ± 1 | 1.6 ± 0.3 | 1.5 ± 0.2 | 1.5 ± 0.7 |
| 18:3ω3 | 1.2 ± 0.4 | 1.2 ± 0.6 | 0.9 ± 0.4 | 1.2 ± 0.4 | 0.6 ± 0.8 | 1.2 ± 0.6 | 1 ± 0.3 | 1.2 ± 0.7 |
| 20:4ω6 | 2.2 ± 0.5 | 1.4 ± 0.5 | 2 ± 0.9 | 2.1 ± 0.3 | 0.7 ± 1 | 2.4 ± 0.2 | 2.1 ± 0.6 | 1.3 ± 0.5 |
| 20:5ω3 | 11.8 ± 3.4 | 9.2 ± 3.2 | 10.9 ± 4.8 | 11.9 ± 2.3 | 5.1 ± 7.2 | 14.1 ± 3.8 | 11.4 ± 2.4 | 8.5 ± 3.2 |
| 22:5ω3 | 2.2 ± 1.5 | 1.5 ± 0.8 | 1.5 ± 0.6 | 1.5 ± 0.2 | 0.7 ± 1 | 2 ± 0.1 | 2.1 ± 1.3 | 1.6 ± 0.9 |
| 22:6ω3 | 27.6 ± 5.8 | 25.4 ± 7.2 | 31.3 ± 8.9 | 26.1 ± 5.8 | 14.5 ± 14.1 | 33.2 ± 4.3 | 31.1 ± 6.1 | 25.6 ± 6.2 |
| ΣPUFA | 47.8 ± 9.2 | 41.4 ± 11.8 | 49 ± 16.1 | 46.1 ± 8.6 | 22.8 ± 25.8 | 55.8 ± 6.7 | 50.7 ± 8.8 | 40.6 ± 10.6 |
| ΣΩ3 | 43.2 ± 8.6 | 37.8 ± 10.8 | 44.9 ± 14.4 | 41 ± 8.2 | 21 ± 23.3 | 50.8 ± 6.3 | 46.4 ± 8.1 | 37.1 ± 9.6 |
| ΣΩ6 | 4.6 ± 1 | 3.6 ± 1.2 | 4.1 ± 1.8 | 4.9 ± 0.6 | 1.8 ± 2.5 | 4.9 ± 0.5 | 4.3 ± 1 | 3.4 ± 1.2 |
| Ω3/ Ω6 | 9.7 ± 2.2 | 9.9 ± 3.1 | 9.3 ± 4 | 8.3 ± 1.4 | 5.3 ± 7.5 | 10.4 ± 0.5 | 11 ± 1.7 | 9.7 ± 3.5 |
| Others* | 1.8 ± 0.1 | 1.8 ± 0.2 | 1.9 ± 0.1 | 2.9 ± 0.1 | 1.1 ± 0.1 | 2.6 ± 0.2 | 2.7 ± 0.2 | 1.8 ± 0.1 |

*17:1, 18:1ω9t, 20:1ω, 18:2ω6t, 18:3ω6, 18:4ω3, 20:2ω6, 20:3ω6, 20:3ω3, 22:4ω6
