# Supplementary material for: Novel signature fatty acid profile of the giant manta ray suggests reliance on an uncharacterised mesopelagic food source low in polyunsaturated fatty acids
Source: PLoS One. 2018 Jan 12;13(1):e0186464. doi: 10.1371/journal.pone.0186464 (PMC5766321; doi:10.1371/journal.pone.0186464)
Supplement: S2 Table — The FA profiles of M. birostris were not significantly different among years (ANOSIM, R = 0.04753, p = 0.27). (PDF) [file pone.0186464.s006.pdf]

Fatty Acid	*M. birostris* 2012 (n = 9)	*M. birostris* 2013 (n = 11)	*M. birostris* 2014 (n = 29)
14:0	6.9 ± 8.7	2.2 ± 2.5	2.1 ± 3.3
16:0	36.9 ± 6	29.6 ± 4.7	25.9 ± 7.3
18:0	21 ± 5.8	25.7 ± 3.7	23 ± 6
20:0	1 ± 0.9	0	1.5 ± 4.2
22:0	0.6 ± 1	0	3.4 ± 5.5
ΣSFA	67.2 ± 6.1	57.5 ± 4.9	56.1 ± 11.6
15:1	0.4 ± 0.5	0.7 ± 1	0
16:1ω7	2 ± 0.9	1.9 ± 1.7	1.8 ± 2
18:1ω9t	0.4 ± 0.8	0	5.2 ± 8.9
18:1ω9c	21.6 ± 4	27.9 ± 4.6	16.4 ± 8.8
18:1ω7	4 ± 1.3	7.6 ± 1.9	4.8 ± 4.3
20:1ω9	0.5 ± 0.6	1.4 ± 1.3	1.1 ± 1.7
22:1ω9	0.4 ± 0.7	0 ± 0	8.7 ± 13.7
24:1ω9	1.6 ± 1.4	0.4 ± 1	0.3 ± 0.9
ΣMUFA	30.9 ± 5.5	40 ± 5.3	38.3 ± 13.1
18:2ω6c	0.3 ± 0.4	0.3 ± 0.7	1.2 ± 2.3
18:3ω3	0	0	0.6 ± 1.9
20:4ω6	1 ± 1.1	1.6 ± 2.4	2.1 ± 2.3
22:5ω3	0	0	0.6 ± 2.4
22:6ω3	0.1 ± 0.4	0.6 ± 1.4	0.7 ± 2.7
ΣPUFA	1.9 ± 1.6	2.5 ± 4.4	5.6 ± 7
ΣΩ3	0.4 ± 0.6	0.6 ± 1.4	1.9 ± 5.6
ΣΩ6	1.5 ± 1.5	1.9 ± 3.1	3.7 ± 3.8
Ω3/ Ω6	0.1 ± 0.2	0.1 ± 0.2	0.2 ± 0.5
Others*	1.1 ± 0.1	0	0.7 ± 0.1

*15:0, 17:0, 19:0, 22:0, 24:0, 14:1ω5, 17:1, 20:1ω7, 18:2ω6t, 18:3ω6, 18:4ω3, 20:2ω6, 20:3ω6, 20:3ω3, 20:5ω3, 22:4ω6
