# Supplementary material for: Novel signature fatty acid profile of the giant manta ray suggests reliance on an uncharacterised mesopelagic food source low in polyunsaturated fatty acids
Source: PLoS One. 2018 Jan 12;13(1):e0186464. doi: 10.1371/journal.pone.0186464 (PMC5766321; doi:10.1371/journal.pone.0186464)
Supplement: S3 Table — Fatty acids with an average contribution >5% are included and data was not transformed prior to analysis. (PDF) [file pone.0186464.s007.pdf]

Fatty Acid	Contribution to similarity (%)	Cumulative contribution to similarity (%)	Cluster A (% of total FA)	Cluster B (% of total FA)
18:1ω9t	24.8	24.8	17.2	0.1
18:1ω9c	21	45.86	8.2	22.7
22:0	10.3	56.2	7.8	0.8
16:0	9.1	65.3	26	31
18:1ω7	8.9	74.3	0	6.2
22:1ω9	7.5	81.8	4.8	4.8
18:0	5	86.8	24.1	24
