# Supplementary material for: Novel signature fatty acid profile of the giant manta ray suggests reliance on an uncharacterised mesopelagic food source low in polyunsaturated fatty acids
Source: PLoS One. 2018 Jan 12;13(1):e0186464. doi: 10.1371/journal.pone.0186464 (PMC5766321; doi:10.1371/journal.pone.0186464)
Supplement: S4 Table — Fatty acids with an average contribution >5% are included and data was not transformed prior to analysis. (PDF) [file pone.0186464.s008.pdf]

Fatty Acid	Contribution to similarity (%)	Cumulative contribution to similarity (%)	Cluster 1 (% of total FA)	Cluster 2 (% of total FA)
16:0	46.9	46.9	0.164	23.1
22:6ω3	18	64.9	34.6	25.9
20:5ω3	9.2	74.1	13.9	9.41
